# *In-vitro* Studies of Anti-EGFR Tyrosine Kinase Activity of Thai nutraceutical Plants

**DOI:** 10.22037/ijpr.2017.2022

**Published:** 2020

**Authors:** Suwanna Semsri, Chanyatorn seatew, Siriluk Rattanabunyong, Sirigade Ruekit, Natharinee Horata, Aussara Panya, Pa-thai Yenchitsomanus, Orathai Sawatdichaikul, Kiattawee Choowongkomon

**Affiliations:** a *Faculty of Medical Ttechnology, Huachiew Chalermprakiet University, Samut Prakarn 10540, Thailand. *; b *Interdisciplinary Graduated Program in Genetic Engineering, Graduate School, Kasetsart University, Chatuchak, Bangkok 10900, Thailand. *; c *Department of Biochemistry, Faculty of Science, Kasetsart University, 50 Ngam Wong Wan Rd, Chatuchak, Bangkok 10900, Thailand. *; d *Division of Molecular Medicine, Department of Research and Development, Faculty of Medicine Siriraj Hospital, Mahidol University, Bangkok, 10700, Thailand. *; e *Department of Biochemistry, Faculty of Medicine Siriraj Hospital, Mahidol University, Bangkok, 10700, Thailand. *; f *Department of Nutrition and Health, Institute of Food Research and Product Development, Kasetsart University, 50 Ngam Wong Wan Rd, Chatuchak, Bangkok 10900, Thailand. *; g *Center for Advanced Studies in Tropical Natural Resources, NRU-KU, Kasetsart University, Chatuchak, Bangkok, 10900, Thailand.*

**Keywords:** Tyrosine kinase, Thai nutraceutical plants, Anticancer, Azadirachta indica, Brucea javanica (L.) Merr

## Abstract

Functional foods have emerged as a new approach to improve human health in term of nutraceutical to prevent people from illness rather than cure patients through medical treatment. In Asian society, particularly in Thailand, the utilizations of functional ingredients have been integrated in every parts of ordinary life. In this study, the tyrosine kinase activity of epidermal growth factor receptor (EGFR) inhibiting properties of 23 Thai’s herbs-ethanol extracts have been examined. The crude extracts of only four species that inhibit the activity of EGFR-tyrosine kinase, *Azadirachta indica *(neem, Sa-dao), *Brucea javanica *(L.) Merr. (Rajadad), *Hibiscus sabdariffa *L. (Roselle, Krachiap daeng), and *Saccharum chinensis *Roxb. (Red sugar cane). Moreover, only ethanol extractions from *A. indica *and *B. javanica *were also showed antitumor effect to non-small cell lung cancer, A549 cells.

## Introductions

Epidermal growth factor receptor (EGFR, ErbB) and its related proteins (ErbB2, ErbB3, and ErbB4) in ErbB family, share both structures and functions. They hold the crucial roles in cell regulations. In addition, they are also relevant to cancer pathogenesis. Unsurprisingly, proteins in this family are popular rational targets for therapeutic intervention (1). Furthermore, aberrant activity of EGFR is observed more than 60% of patients with non-small cell lung cancer (NSCLC), the largest subset of lung cancer and the major cause of cancer death (2). Several reasons as mentioned, therefore, the EGFR protein has been emphasized in various research fields, especially in cancer research area. A number of strategies influencing this receptor, and its downstream signal cascades, including monoclonal antibodies, small molecule tyrosine kinase inhibitors, antisense oligonucleotides inhibiting EGFR synthesis and antibody-based immunoconjugates, have been evaluated. Among various types of EGFR inhibiting agents, small molecule tyrosine kinase inhibitor is established clinical activity and regulatory approval for the treatment of cancer. The anti-cancer drugs, gefitinib (Irressa® Astra Zeneca), erlotinib (Tarceva® Genetech), lapatinib (Tykerb® GlaxoSmithKline), and vandetanib (Astra Zeneca) have been approved to use for non-small cell lung cancer (NSCLC) treatment in 2003 (gefitinib) and 2004 (erlotinib), breast cancer in 2007 (lapatinib) and thyroid cancer in 2011 (vandetanib), respectively. Moreover, many of them, pelitinib, neratinib, and afatinib are under investigation both in clinical trial phase I to III, and enzymatic assay. 

Even though the modern medicines are very successful in past decades, the alternative medicines that endowed with the naturally attitude are still popular (wildly used). Currently, people pay attention to phytonutrients as “nutraceutical” which provides health and medical benefits with the new concept from a food and food product. Generally, folk or local food (slow cooking) contains many natural compounds, which not only cure but also prevent from suffering some symptoms (or diseases) and promote health. This motivates the research to look for the more effective EGFR tyrosine kinase inhibitors from natural products for using in the cancer treatment and preventing. That is why people today look back into the ways of nature. Furthermore, there are tons of scientific studies report about the properties of natural product of phytonutrient in several chronic diseases (3-8), endowed with our previous prediction of the potent compounds from herbs inhibit the kinase domain of epidermal growth factor receptor, EGFR-TK (9).

This present study therefore investigated inhibitory effects of crude extracts from a set of medicinal herbs against EGFR-TK activity both enzymatic inhibition and cell line toxicity studies. The known anti-EGFR-TK drug, gefitinib was included in this study as the positive control. Dimethyl sulfoxide (DMSO), the chemical solvent, was used as the negative control.

## Methods

*Preparation of plant materials*

Twenty-three Thai’s medicinal plants were purchased from a folk-medicinal store in Bangkok, Thailand. Scientific and Thai local names as well as the used part (s) of medicinal plants are showed in Table 1. All of dried plant materials were extracted successfully by 95% ethanol. 100 g of each plant material was soaked with 500 mL of 95% ethanol in Erlenmeyer flask with covered lid for 72 h at room temperature. Then, each supernatant was filtered with 10 nm Whatman filter paper. The filtrate was concentrated by a rotary evaporator under 50 °C. The evaporated weights of each sample were recorded. Each extract was dissolved in 95% ethanol to produce a stock solution at concentration 20 mg/mL and then, they were kept at -20 ºC until further require. 

The extract yield (%w/w) was determined from all extracts by using the formular:

$\mathrm{Yield}(\%)=\frac{\mathrm{The weight of evaporated extract}}{\mathrm{The weight of dried plant}}\times 100$

Equ.1

The extractions of crude herbs by ethanol were diluted to 6 mg/mL in 1X kinase buffer (50 mM Tris-HCl, 20 mM MgCl2, 1 mM EGTA, 0.01% Brij 35, pH 7.5) containing 2 mM DTT. The crude extracts were kept at 4 °C prior to use. 

*Tyrosine kinase inhibition assay*

Kinase domain of EGFR, active, was purchased from Millipore Corporation, Billerica, MA, USA. Measurements of tyrosine kinase inhibition assay by using fluorescence were performed as its descriptions. The procedures contain following steps; (i) enzyme-ligand incubation; 12.5 µL of 12 nM tyrosine kinase enzyme were incubated with 12.5 µL of 100 µg/mL each herbal extracted sample at room temperature for 5 min. (ii) Anti-phosphotyrosine fluorescein coupling reaction; 20 µL of antibody Beacon detection complex (6.67 nM Anti-phosphotyrosine antibody, 3.3 nM Oregon Green 488 ligand, 0.027 mg/mL poly (Glu:Tyr)) were added into pre-incubated enzyme with each herbal extracted sample. The mixed solutions were incubated at room temperature absent of light for 10 min. (iii) The activation and monitoring the reactions, each reaction was started via adding 30 µL of 1.25 mM ATP. The signal has been measured by using a fluorescence microplate reader (Tecan, Infinite F200 PRO) with excitation and emission wavelength of 485 and 530 nm, respectively. Three independent experiments have been performed.

Table 

*Fifty percent inhibitory concentration (IC*_50_ *) determinations*

Crude herbal extracts from 95% ethanol were diluted in two-folded dilutions with starting concentration of 4000 µg/mL to final concentration of 7.8 µg/mL. The activity of each extract towards EGFR-TK was investigated by IC_50_ measurements using the same protocol of TK assay as described in previous part. Subsequently, each reaction was activated, and the signal was monitored as mentioned in previous method. Nonlinear regression dose-response curves were plotted against activity and log value of inhibitor’s concentration. The GraphPad Prism program (GraphPad Software Inc., San Diego, CA, USA) has been performed to calculate the fifty percent inhibitions (IC_50_) of each sample.

*Cell culture*

In this study, two types of cell lines were used to investigate the effect of crude extracts. First, the peripheral blood mononuclear cells (PBMC) were used as a representative of normal human cell. Second, A549 cells are adenocarcinomic human alveolar basal epithelial cells, which overexpress EGFR. PBMC cells were isolated from whole blood, which was obtained from healthy people, by density gradient centrifugation using Lymphoprep (Axis-shield, UK). PBMC Cells were seeded into 96 well tissue culture plates with 1x10^5^ cells per 100 µL of media in each well. A549 cells were cultured at 37 °C, 5% CO_2_ incubator in Dulbecco’s Modification of Eagle’s Medium (DMEM) with L-glutamine supplemented with 10% fetal calf serum (FCS), 1× non-essential amino acids (NEAA), 1 mM sodium pyruvate and antibiotic (100 µg/mL penicillin and 10 µg/mL streptomycin) (Gibco, USA). A549 were seeded in 96-well plate at 1.5 x10^4^ cells/well in growth media to achieve 80% confluence.

*Cell viability assay (MTT assay)*

After the cultivation of PBMC cells and A549 cell lines, the crude herbal extracts were processed as described by (10). The cultured plates were incubated overnight. The cytotoxicity of herb extracts on PBMC was detected by MTT [3-(4,5-dimethylthiazol-2-yl)-2,5-diphenyltetrazolium bromide] assay (Sigma-Aldrich, MO, USA) as described previously (11).

**Figure 1:**
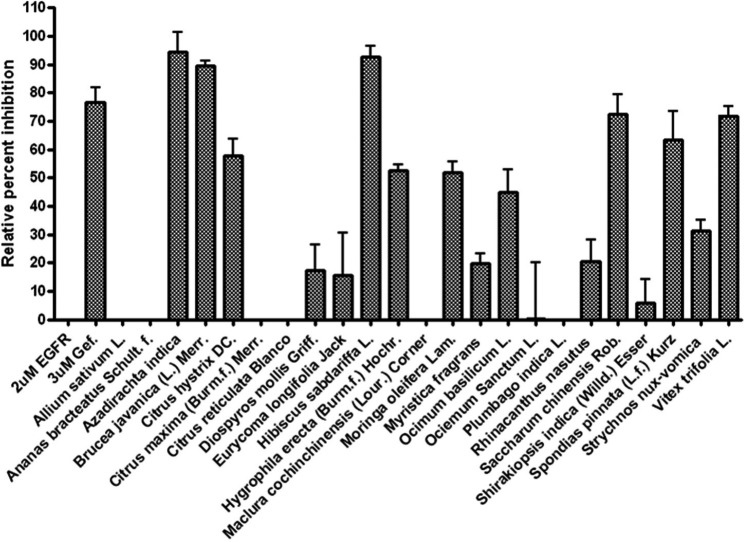
Percentage of inhibitory from ethanol extracted herbal

**Figure 2:**
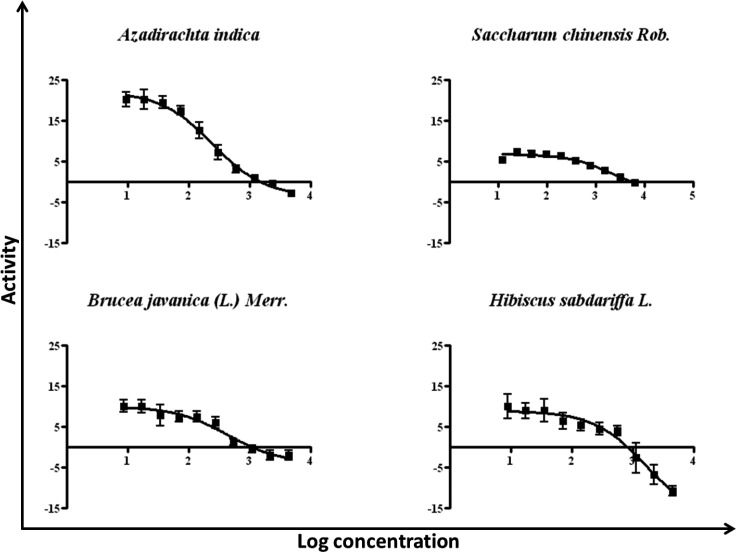
Diagram of IC_50_ of 95% ethanol crude extracts from 5 Thai medicinal plants to EGFR-TK

**Table 1:** Selected Thai medicinal plants, the parts used, 95% ethanol extraction yields

No.	Scientific Name	Part Used	Yield (%)	Thai's name	Common name			
			95% Ethanol					
1	*Allium sativum *L.	Bulb	0.997	Kra thiam	Garlic			
2	*Ananas bracteatus *Schult. f.	Fruit bark	49.240	Supparod				
3	Azadirachta indica*	Leaves	5.382	Sa-dao	Neem Tree			
4	*Brucea javanica *(L.) Merr.*	Fruit	4.919	Rajadad				
5	*Citrus hystrix *DC.	Fruit bark	28.050	Ma-Grude				
6	*Citrus maxima *(Burm.f.) Merr.	Fruit bark	2.170		Pomelo, Shaddock			
7	*Citrus reticulata *Blanco	Fruit bark	17.465		Mandarin orange, Tagerine orange			
8	*Diospyros mollis *Griff.	Stem	16.983	Ma-Glear				
9	*Eurycoma longifolia* Jack	Stem	2.649	Pla lai phueak (Central)				
10	*Hibiscus sabdariffa *L.*	Flower	16.887	Krachiap daeng	Roselle, Jamaican Sorrel			
11	*Hygrophila erecta *(Burm.f.) Hochr.	Stem	8.072	Toi-ting				
12	*Maclura cochinchinensis *(Lour.) Corner	Stem	14.232					
13	*Moringa oleifera *Lam.	Seed	2.997	Ma-rum	Horse radish tree			
14	*Myristica fragrans *	Stem	8.010	Janntead	Nutmeg Tree			
15	*Ocimum basilicum *L.	Leaves	12.458	Horaphaa (General)	Sweet Basil			
16	*Ociemum Sanctum *L.	Leaves	15.829	Kraprao-daeng	Holy Basil, Tulsi, Tulasi, Kemangen.			
17	*Plumbago indica *L.	Root	20.033	Chetta mun phloeng daeng				
18	*Rhinacanthus nasutus *	Aerial part	6.307	Thong pun chung				
19	*Saccharum chinensis *Roxb.*	Stem	2.035	Aoidang	Red sugar cane			
20	*Shirakiopsis indica *(Willd.) Esser	Fruit	1.267	Samor tale				
21	*Spondias pinnata *(L.f.) Kurz	Fruit	3.352	Ma kok				
22	*Strychnos nux-vomica *	Seed	1.329	Salang Jai	snake wood			
23	*Vitex trifolia *L.	Root	5.705	Khon thi so				

*the high inhibiting activity against EGFR-TK

**Table 2:** IC50 values of 5 Thai's medicinal plants against tyrosine kinase activity of EGFR

	**Name**	**IC**_50_ ** (Mean ± SEM)**
		**(µg/mL)**
	*Azadirachta indica*	207.7 ± 9.247
	*Brucea javanica *(L.) Merr.	369.9 ± 4.856
	*Hibiscus sabdariffa *L.	1477 ± 7.129
	*Saccharum chinensis Rob.*	1270 ± 2.555

**Table 3:** Antiproliferative activity of 4 Thai edible herbs extract on A549 cell line expressed as IC50a (µg/mL) values

**Name**	**IC**_50_ ** (Mean ± SEM)**	***P value***
	**(µg/mL)**	
*Azadirachata indica*	78.6 ± 3.1	*P *< 0.05
*Brucea javanica *(L.) Merr.	19.8 ± 1.2	*P *< 0.05
*Saccharum chinensis *Roxb.	>1000	*P *> 0.05
*Hibiscus sabdariffa* L.	>1000	*P *> 0.05
		

a The IC50 is the concentration of the plant material at which 50% inhibition of tumor activity (EGFR overexpression) was observed. The lower of the IC50 number that the greater overall effectiveness of the material in question. The results showed mean ± standard deviation based on triplicate analyses.

## Results and Discussion

*Effects of crude extract of edible herbs on the activity of EGFR-TK*

Twenty three kinds of edible herbs are collected from traditional pharmacies (medicinal herb shop) as well as local markets around Bangkok, Thailand. In Asian countries, particularly South-east Asia, these plants are ordinary used as ingredient in not only the traditional remedies but also in cuisines. These Thai’s edible plants are consumed as the vegetables along with chili paste, juices, or as ingredients in the various kinds of soups and other fantastic Thai’s dishes, both foods and sweets including beverages. Since there are abundant of phytochemical compounds accumulating in parts of these phytonutrient-herbs, the local Thai dished also provide plenty of nutraceutical to strengthen people health. In this study, we aimed to investigate the inhibitory effects of the 95% ethanol extracts from edible herbs against activity of EGFR-TK. All details of each sample which describing scientific name, common name, local name, part of used, and % yield extracts were elucidated in Table 1. In order to examine the ability of all crude extracts to inhibit the EGFR-TK enzyme activity, tyrosine kinase inhibitory assay has been performed. The results of these observations were reported in term of *relative percent inhibition *values as present in the bar graph (Figure 1). The EGFR-TK known inhibitor, gefitinib (at concentration 3 µM) was used as the positive control in this screening. Four species from all twenty-three Thai’s edible herbs, present the *relative percent inhibition *values against EGFR-TK higher than others. Those herbs are *Azadirachta indica *(neem, Sa-dao), *Brucea javanica *(L.) Merr. (Rajadad), *Hibiscus sabdariffa *L. (Roselle, Krachiap daeng), and *Saccharum chinensis *Roxb. (Red sugar cane). Nevertheless, other herbs possessed slightly inhibitory activities against enzyme tyrosine kinase of EGFR. Surprisingly, the extracts of general edible plants that were believed to present many nutraceutical properties such as garlic (*A. sativum* L.), pine-apple (*A. bracteatus* Schult. f.) kinds of oranges (*C. maxima *(Brm.f.) Merr and *C. reticulate* Blanco) and from other two local regional species *M. cochinchinensis *(Lour.) Corner, and *S. indica* (Willd.) Esser, lacked of effect to the activity of the kinase enzyme of EGFR. The second group, nutrient plants possessed moderate inhibitory activities on EGFR-TK composed of kaffir lime (*C. hystrix* DC), Toi-ting (*H. erecta* (Burm.f.) Hochr.), horse radish tree (*M. oleifera* Lam.), sweet basil (*O. basilicum* L.), and Khon-thi-so (*V. trifolia* L.). In addition, the mild effects on the kinase domain of EGFR consist of seven species, which are the rest species reported in Figure 1. 

According to the *relative percent inhibition *values of these, in particular four plants, for further precision fifty percent inhibitory concentration (IC_50_) determinations of EGFR-TK activity have been performed. The activity of tyrosine kinase enzyme versus log concentration of each extracts presented in Figure 2 and concluded the values in Table 2. These data indicated that the crude extracts from *A. indica* and *B. javanica* possessed the abilities to inhibit EGFR-TK better than from *H. sabdariffa* and *S. chinensis* in range of 3 to 7 folds. Notably, the crude extracts from these two latter did not significantly inhibit EGFR-TK as illustrated in the *relative percent inhibition.*

*The cytotoxicity and viability of crude extracts to the NSCLC and normal cells*

To examine the efficiency of these crude samples could inhibit the viability of lung cancer cell lines; the MTT assay was performed on adenocarcinomic human alveolar basal epithelial cells (A549). The A549 cell line is the representative of the non-small cell lung cancer type, which showed overexpression of EGFR and is sensitive to EGFR inhibitors. Our results indicated that both crude extracts from *A. indica *and *B. javanica* showed lower IC_50_ against A549 than *H. sabdariffa *and *S. chinensis* which lacked of inhibiting activity on the A549 cell line at our tested concentration (Table 3). In particular, the crude extract from *B. javanica *presented inhibitory effect on EGFR-TK stronger than from *A. indica* about 4 times (Table 3).

Moreover, to confirm the cytotoxicity effects of four extracted solutes to normal human cells, peripheral blood mononuclear cells (PBMC) have been cultured to evaluate these crudes (Supplementary Figure 1). The results of toxicity test indicated that the crude extracts of all four herbs were no affect to normal cells at the effective concentration against cancer cells. Interestingly, the extracts of both *A. indica* and *B. javanica*, are generally known to have anti-cancer activities (4, 12-14). In addition, parts of these two plants are ingredients of the traditional medicine remedies in several countries. The crude samples of these two mentioned plants presented the potential to inhibit the viability of the NSCLC cells and slightly affect to the cytotoxicity of the normal cells (PBMC). The extracted samples of other two species, generally consumed as juice, showed no effects both A549 and PBMC cells. *B. javanica* and *A. india* were used for traditional medicine to treat malaria, dysentery and cancer (15, 16). This study showed neither *B. javanica* nor *A. india *had any effect on PBMC, normal cell. 

Plants are plenty of chemo-preventive agents that show anti-carcinogenic properties to inhibit cancer cells. Several commercial cancer drugs are developed from this source. These reasons, therefore, emphasized the intense interest on medicinal herbs for finding promising anti-cancer drug. Several researches have tried to find anti-cancer against cancer cells line without focusing on mechanism of this inhibition. Recently, Wang *et Al*. have tested crude extracts of Chinese herbs against EGFR-kinase (17). The five ethyl acetate extracts, *Artemisia argyi, Lonicera macranthoides, Spatholobus suberectus, Curcuma longa*, and *Galla chinensis*, and the ethanol extract of *Eriobotrya japonica* possessed EGFR inhibitory activities.

Four selected plants are tropical tree, shrub, ivy, and herbaceous plant, which commonly found in South and South-East Asia, including Thailand. These plants are ingredients of everyday food in Asian society.* A indica* (Sa-dao or neem) is generally serve accompany with chili paste in the food set. Furthermore, the result of this investigation is also corresponds with our previous computational studied (9) that the crude extracted from *Azadirachata indica* possesses high inhibitory activity to kinase domain of EGFR. More than sixty active compounds from neem have been identified which is isolated from all over parts of neem, leaves, stem, bark, and root. At least two active compounds, 1,3-diacetylvilasinin (18) and 28-deoxonimbolide (19) exhibited the cytotoxicity against NSCLC cells, human prostate (PC-3), pancreatic (PACA-2) cell and HL60 leukemia cells in µM range.

*Brucea javanica* (Rajadad) is used in various recipes, in Thai, use dried fruit to reduce phlegm, nourishing bile and cure Tan Sang toxic. Furthermore, in Chawa; Indonesia, seed of *B. javanica *is used for the treatment of intestinal diseases. In Philippines, fresh fruit is used to cure stomachache while in China, *B. javanica *is known as chelation worms in the stomach and cure dysentery. The common compounds found in *B. javanica* are classified in the group of brucamarine. The phytochemical from *B. javanica* is spotted light, according to it has shown impressive efficacy for treating various diseases including cancer. Bruceine D, a quassinoid compound extracted from *B. javanica*, showed anticancer effect against NSCLC and pancreatic cancer cells, besides bruceanols D, E and F exhibited cytotoxicity against various types of human cancers, malignant melanoma (RPMI-7951), lung carcinoma (A-549), ileocecal adenocarcinoma (HCT-8), epidermoid carcinoma of the nasopharynx (KB), and medulloblastoma (TE-671), and against murine lymphocytic leukemia (P-388) (13, 14, 20, 21). 

From these results, there are two distinguish herbs showed the inhibitory activity to the A549 cancer cell line, which are the crude extract from *B. javanica* and *A. indica*. Nevertheless, the other two nutraceutical herbs, *H. sabdariffa* and *S. chinensis* lacked of the inhibitory effect toward NSCLC, A549 cancer cell even though they showed the strong inhibitory activity against EGFR-TK. The phytochemical compounds of these two latter herbs might be able to penetrate cell membrane. 

## Supporting Online Material

Supplementary Material
