# Supplementary material for: In-vitro Studies of Anti-EGFR Tyrosine Kinase Activity of Thai nutraceutical Plants
Source: Iran J Pharm Res. 2020 Spring;19(2):199–206. doi: 10.22037/ijpr.2017.2022 (PMC7667552; doi:10.22037/ijpr.2017.2022)
Supplement: Supplementary Material [file ijpr-19-199.s001.pdf]

Supplementary Materials for

In-vitro Studies of Anti-EGFR Tyrosine Kinase Activity of Thai nutraceutical Plants

Suwanna Semsri, Chanyatorn seatew, Siriluk Rattanabunyong, Sirigade Ruekit, Natharinee Horata[a], Aussara Panya, Pa-thai Yenchitsomanus , Orathai Sawatdichaikul[*] and Kiattawee Choowongkomon*

*To whom correspondence should be addressed

ifrots@ku.ac.th , fsciktc@ku.ac.th

Volume 19, Issue 2 (Spring 2020)

This PDF file include

Figure s1

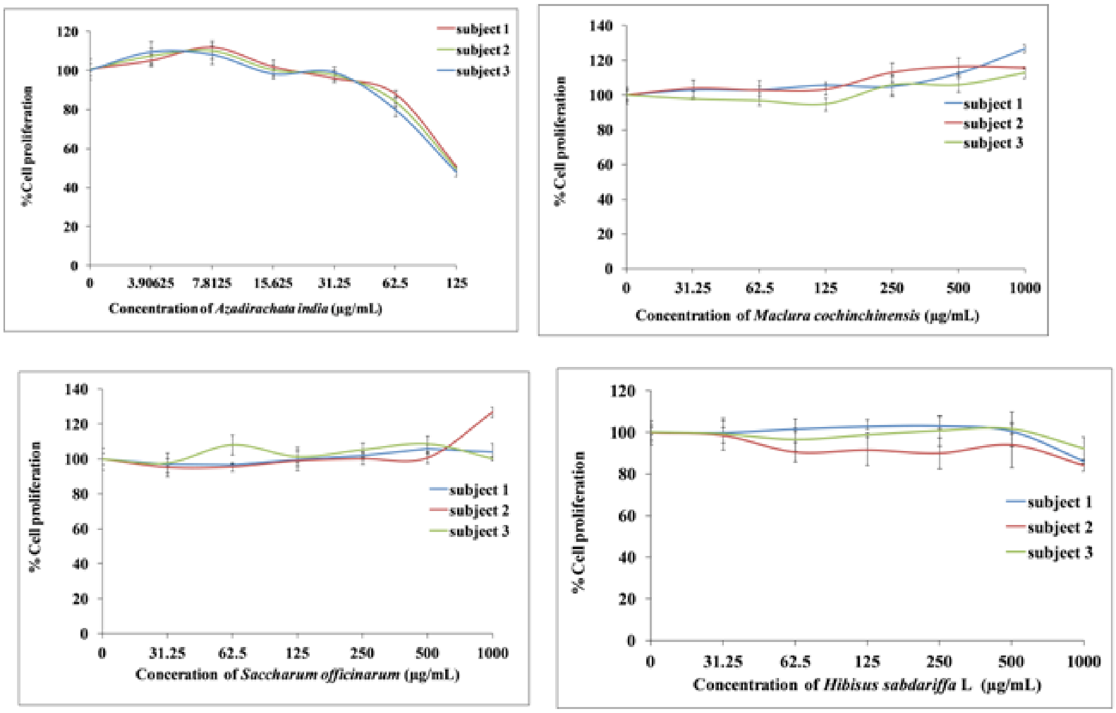

Supplementary Figure 1 The cytotoxicity of four herb extracts on PBMC was detected by MTT
